# High-Resolution Scanning Coded-Mask-Based X-ray Multi-Contrast Imaging and Tomography

**DOI:** 10.3390/jimaging7120249

**Published:** 2021-11-24

**Authors:** Zhi Qiao, Xianbo Shi, Michael Wojcik, Lahsen Assoufid

**Affiliations:** Advanced Photon Source, Argonne National Laboratory, Lemont, IL 60439, USA; mwojcik@anl.gov (M.W.); assoufid@anl.gov (L.A.)

**Keywords:** X-ray phase-contrast imaging, speckle tracking, coded phase mask, multi-contrast tomography

## Abstract

Near-field X-ray speckle tracking has been used in phase-contrast imaging and tomography as an emerging technique, providing higher contrast images than traditional absorption radiography. Most reported methods use sandpaper or membrane filters as speckle generators and digital image cross-correlation for phase reconstruction, which has either limited resolution or requires a large number of position scanning steps. Recently, we have proposed a novel coded-mask-based multi-contrast imaging (CMMI) technique for single-shot measurement with superior performance in efficiency and resolution compared with other single-shot methods. We present here a scanning CMMI method for the ultimate imaging resolution and phase sensitivity by using a coded mask as a high-contrast speckle generator, the flexible scanning mode, the adaption of advanced maximum-likelihood optimization to scanning data, and the multi-resolution analysis. Scanning CMMI can outperform other speckle-based imaging methods, such as X-ray speckle vector tracking, providing higher quality absorption, phase, and dark-field images with fewer scanning steps. Scanning CMMI is also successfully demonstrated in multi-contrast tomography, showing great potentials in high-resolution full-field imaging applications, such as in vivo biomedical imaging.

## 1. Introduction

Hard X-ray imaging has played an essential role in modern material, biomedical, and physical research. As one of the foremost imaging techniques, computed tomography (CT) has been widely used in visualizing biological tissue with three-dimensional information owing to the large penetration depth and non-destructive property of hard X-rays. Initially, CT was based on absorption radiography, such as the CT scan in medical applications. Since X-rays are much more sensitive to the sample phase than absorption, phase-contrast imaging and tomography have generated increased interest in studying biological samples, especially soft tissues [1].

Various phase-contrast imaging methods have been developed, such as propagation-based techniques [2], grating interferometry [3,4,5,6], and speckle tracking [7,8,9,10]. Among these methods, grating interferometry and speckle tracking have been used in various applications, such as phase-contrast and dark-field imaging, and tomography, wavefront sensing, and at-wavelength metrology [11,12,13,14,15,16,17,18,19,20] due to their quantitative measurements of absorption, phase, and dark-field signals. Compared with grating interferometry, speckle tracking has better spatial resolution and phase sensitivity, and has a more flexible implementation, thus allowing a wider range of applications.

Speckle tracking methods are based on the near-field speckle propagation process, and the resulting differential phase can be reconstructed by analyzing the speckle pattern displacement. Many phase reconstruction methods have been developed, such as cross-correlation-based methods [20,21,22], the transport of intensity equation (TIE) [9], and optimization-based analysis [10]. The TIE-based process can recover the differential phase analytically with a single-shot measurement, but extra sample assumptions and pre-knowledge are required for the quantitative reconstruction. The cross-correlation-based X-ray speckle tracking (XST) and optimization-based unified modulated pattern analysis (UMPA) can be used to reconstruct the phase and dark-field images simultaneously. However, small patch analysis with a sub-window is necessary to find the exact speckle pattern displacement. The choice of sub-window size restricts the available phase sensitivity and dynamic range. Otherwise, more speckle scanning positions are required for the data analysis, such as in the X-ray speckle vector tracking (XSVT) method [23]. In addition, most speckle tracking methods use random speckle generators, such as sandpaper and membrane filters, which provide limited speckle pattern contrast. Moreover, realizing a practical pre-calibration to achieve reference-free measurement is challenging.

A new coded-mask-based multi-contrast imaging (CMMI) has been proposed more recently [24] to achieve pixel-wise speckle displacement analysis without using a sub-window. Instead of a sandpaper-type speckle generator, a predesigned binary phase mask is used to attain higher-contrast speckle patterns. The single-shot version of the CMMI method has already been demonstrated with superior performance compared to other single-shot speckle-tracking methods. By combining with deep learning, single-shot CMMI aims to advance fast imaging and wavefront sensing applications [24]. In this work, we present a scanning CMMI method towards the ultimate resolution and phase sensitivity. To achieve this goal, we combine the use of coded masks, the flexible scanning mode, the most advanced maximum-likelihood optimization algorithm, and the multi-resolution analysis procedure. The multi-contrast imaging performance of scanning CMMI is compared with the XSVT method, showing a higher image quality with fewer scanning positions, significantly reducing the experimental complexity and time. Finally, scanning CMMI is demonstrated in tomography measurement, providing high-resolution 3D phase, dark-field, and absorption visualization of a biological sample.

## 2. Methods

### 2.1. Experimental Setup

**Figure 1:**
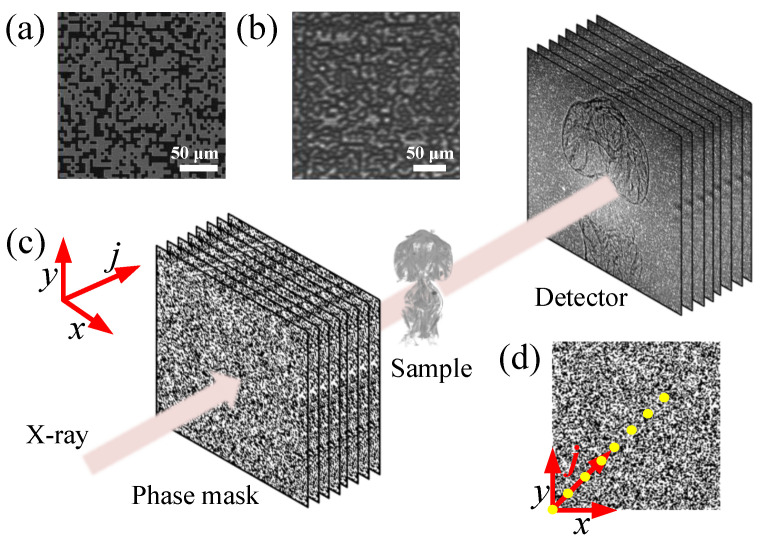
(**a**) SEM image of an example coded phase mask. (**b**) X-ray transmission image of a coded phase mask. (**c**) Schematic of the scanning CMMI experimental setup. (**d**) Diagram showing the linear scanning pattern of the coded phase mask along its diagonal direction. Note that the spacing between dots in (**d**) is not to the actual scale.

The experimental setup at the 1-BM beamline of the Advanced Photon Source (APS) is shown in Figure 1. A coded mask was illuminated by the X-ray beam with a photon energy of 14 keV and energy bandwidth of $10^{-4}$ set by a Si(111) double-crystal monochromator. The coded mask is a binary phase mask made of electroplated square-shaped Au islands with a bit size of 5 µm and a thickness of 2 µm on a Si${}_{3}$N${}_{4}$ membrane substrate. The predesigned binary mask pattern follows a uniform random distribution with an Au occupation of 50%. Fabrication details of the mask can be found in Ref. [24]. The SEM image and X-ray transmission image of the coded phase mask are shown in Figure 1a,b, respectively, as examples. The test sample (an ant) was located downstream of the phase mask, followed by a detector system consisting of a 50 µm thick LuAG:Ce scintillator, a 45° visible-light reflecting mirror, a 10× magnification objective, a tube lens, and an Andor Neo sCMOS camera. The sample-to-detector distance, *d*, is 0.628 m. As a full-field technique, the CMMI field of view (FOV) is determined by the size of X-ray beam and detector. Since the bending magnet source can provide a near-parallel beam with a large size, the detector system limits the imaging FOV to 1.404 × 1.664 mm with a pixel size of 0.65 µm. The scanning CMMI was performed by recording images at *N* different mask positions moving in the transverse plane (x,y). The scanning positions (indexed in *j* and 1≤j≤N) were chosen to be along the diagonal direction of the mask, as shown in Figure 1d, with a uniform step size of 3 µm. The step size was chosen to be smaller than the coded mask bit size and larger than the detector pixel size. The X-ray flux at the sample location is estimated to be around $6\times 10^{9}$ photons/s/mm${}^{2}$, which gives an average count rate of 6 k per pixel on the detector with an exposure time of one second.

### 2.2. Scanning CMMI

The reference image stack contains *N* intensity profiles, $I_{r}^{j}$ at each mask scanning position *j*, without a sample in the X-ray beam. When inserting the sample in the beam, each sample image, $I_{s}^{j}$, is distorted from $I_{r}^{j}$ by the sample’s transmission (*T*), phase (ϕ), and scattering (*D*), given by [24],

(1) $$I_{s}^{j}\left(x+\delta x,y+\delta y\right)=\frac{T(x,y)}{C(x,y)}\{{\bar{I}}_{r}\left(x,y\right)+D\left(x,y\right)[I_{r}^{j}\left(x,y\right)-{\bar{I}}_{r}\left(x,y\right)]\},$$

where ${\bar{I}}_{r}$ is the average intensity of the reference image. $C\left(x,y\right)=1+\frac{\lambda d}{2\pi }\nabla^{2}\phi \left(x,y\right)$ takes into account the local curvatures of the sample phase modulation at the detector location, where λ is the X-ray wavelength. Equation (1) is a generalized formula of TIE by considering both the projection and defocusing effects. The local speckle pattern displacement induced by the sample phase is given by,

(2) $$\delta x=\frac{\lambda d}{2\pi }\frac{\partial \phi (x,y)}{\partial x},\;\delta y=\frac{\lambda d}{2\pi }\frac{\partial \phi (x,y)}{\partial y}.$$

Similar to the single-shot CMMI [24], the transmission (*T*), phase (ϕ), and dark-field (or scattering, *D*) images can be reconstructed using maximum-likelihood optimization. Considering that the measured images are dominated by the Poisson noise, the total cost function can be expressed as the sum of the negative log-likelihood of images at all *N* scanning positions,

(3) $$L_{p}=\sum_{j=1}^{N}\sum_{l,m}\left|I_{s}^{j}\left(T,\phi ,D\right)-{\tilde{I}}_{s}^{j}\;\log\left[I_{s}^{j}\left(T,\phi ,D\right)\right]\right|,$$

where ${\tilde{I}}_{s}^{j}$ is the measured sample image at the scanning position *j*, $I_{s}^{j}\left(T,\phi ,D\right)$ is the calculated sample image based on Equation (1), and *l* and *m* are the pixel indices in the image plane. In order to suppress the reconstruction noise, the total variation regularization $L_{v}\left(f\right)=\sum_{l,m}{\left(|f_{l+1,m}-f_{l,m}\right|}^{2}+|f_{l,m+1}-f_{l,m}{{|}^{2})}^{1/2}$ is introduced into the cost function to provide extra constraints. Then the cost function with regularization is given by [24],

(4) $$L_{c}=L_{p}+\alpha L_{v}{\left(||\nabla D|\right|}_{2})+\alpha L_{v}{\left(||\nabla T|\right|}_{2})+\beta L_{v}\left(\nabla^{2}\phi \right),$$

where α and β are the weight factors and $\left|\right|\cdot {\left|\right|}_{2}$ denotes the square norm. By minimizing Equation (4) using the RMSprop nonlinear optimization method, sample *T*, ϕ, and *D* images can be reconstructed simultaneously.

In case the sample phase ϕ covers a larger dynamic range, for example, a refractive lens with a large radius of curvature and at the same time micron-level voids, accurate determination of the speckle displacement can be challenging. Therefore, we implemented the multi-resolution analysis to reconstruct *T*, ϕ, and *D* images from coarse to fine resolution. The multi-resolution process includes the following steps: (Step 1) Generate pyramid resolution data [$S_{i}\left(I_{s}^{j}\right)$, $S_{i}\left(I_{r}^{j}\right)$] from the sample and reference image stacks, respectively, where $S_{i}$ represents a down-sampling process with a factor of $2^{i}$ in each of the *x* and *y* directions. (Step 2) Obtain $T_{i}$, $\phi_{i}$, and $D_{i}$ from the down-sampled data $S_{i}\left(I_{s}^{j}\right)$ and $S_{i}\left(I_{r}^{j}\right)$ by minimizing the cost function in Equation (4). (Step 3) Up-sample $T_{i}$, $\phi_{i}$, and $D_{i}$ to the next higher resolution level images $T_{i-1}$, $\phi_{i-1}$, and $D_{i-1}$. (Step 4) Use the up-sampled $T_{i-1}$, $\phi_{i-1}$, and $D_{i-1}$ images as initial guesses for the next resolution level (i−1). (Step 5) Repeat steps 2~4 until the recovery of $T_{0}$, $\phi_{0}$, and $D_{0}$ in the original image resolution level.

## 3. Results

### 3.1. Scanning vs. Single-Shot CMMI

**Figure 2:**
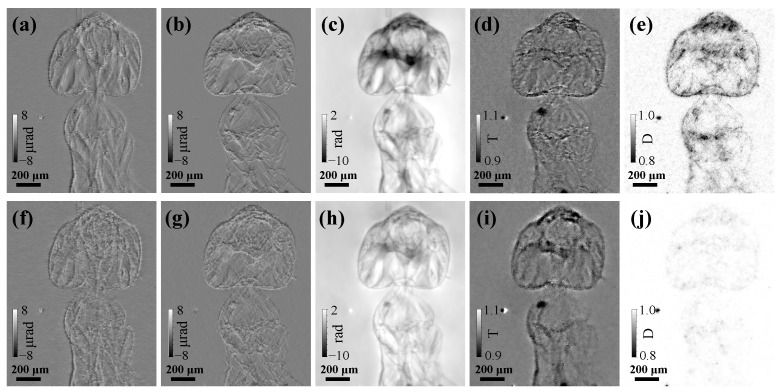
Reconstructed (**a**,**f**) horizontal differential phase ($\phi_{x}$), (**b**,**g**) vertical differential phase ($\phi_{y}$), (**c**,**h**) phase (ϕ), (**d**,**i**) transmission (*T*), and (**e**,**j**) dark-field (*D*) images using (top row figures **a**–**e**) scanning CMMI with *N* = 10 scan positions and (bottom row figures **f**–**j**) single-shot CMMI with *N* = 1 and *j* = 0. Note that the bright spot next to the ant comes from defect of the scintillator.

Figure 2 compares the reconstructed horizontal differential phase ($\phi_{x}$), vertical differential phase ($\phi_{y}$), phase (ϕ), transmission (*T*), and dark-field (*D*) images of the ant sample obtained by scanning CMMI with *N* = 10 scanning positions and compared with results using single-shot CMMI with *N* = 1 using the *j* = 1 images only. For both scanning and single-shot CMMI, the multi-resolution analysis up to the level of *i* = 2 was used. The weight factors used in Equation (4) were α = 0.1 and β = 5, respectively, for the total variation regularization process to suppress noise. A total iteration of 300 was performed to minimize the loss function until convergence for all analyses.

In general, scanning CMMI [Figure 2a–e] improves multi-contrast image quality significantly with much less noise than single-shot CMMI [Figure 2f–j], especially for the phase and dark-field images. Since single-shot CMMI can be treated as a special case (single step) of scanning CMMI, they can share the same experimental setup. The trade-off is then only between the radiation dose (or measurement time) and image quality.

The transmission images in Figure 2d,i show much lower contrast compared with the phase images in Figure 2c,h because of the weak absorption of biological soft tissues. As complementary information, the dark-field scattering images in Figure 2e,j show higher contrast near the sample edge and in high-scattering areas where the phase-contrast could be low. Thus, the multi-contrast imaging capability of the scanning CMMI is successfully demonstrated here.

### 3.2. Scanning CMMI vs. XSVT with Limited Scanning Positions

In this section, we compare the performance of scanning CMMI with the most popular scanning-based speckle-tracking method, X-ray speckle vector tracking (XSVT) [23]. XSVT was developed to improve the resolution of single-shot X-ray speckle tracking, but still with a relatively smaller number of scan positions than the X-ray speckle scanning method [23]. In XSVT, each pixel on the reference image is expanded to a 1D vector with values from the *N* scanning positions. The displacement of the reference pixel induced by the sample is obtained by finding the pixel with the most similar vector on the sample image, which gives the maximum 1D cross-correlation value. The accuracy of the pixel searching process relies heavily on the number *N*. It is straightforward to predict that a small *N* value (e.g., <10) will give a high uncertainty in the 1D cross-correlation process of XSVT and poor image reconstruction quality.

On the other hand, scanning CMMI uses the maximum likelihood optimization of all images directly. Additional mask positions improve the statistics of the cost function minimization, as shown in Equation (3). Therefore, we expect scanning CMMI to work with even very small *N*. One can easily optimize the trade-off between the experimental time and data volume (linearly proportional to *N*) and image reconstruction quality.

**Figure 3:**
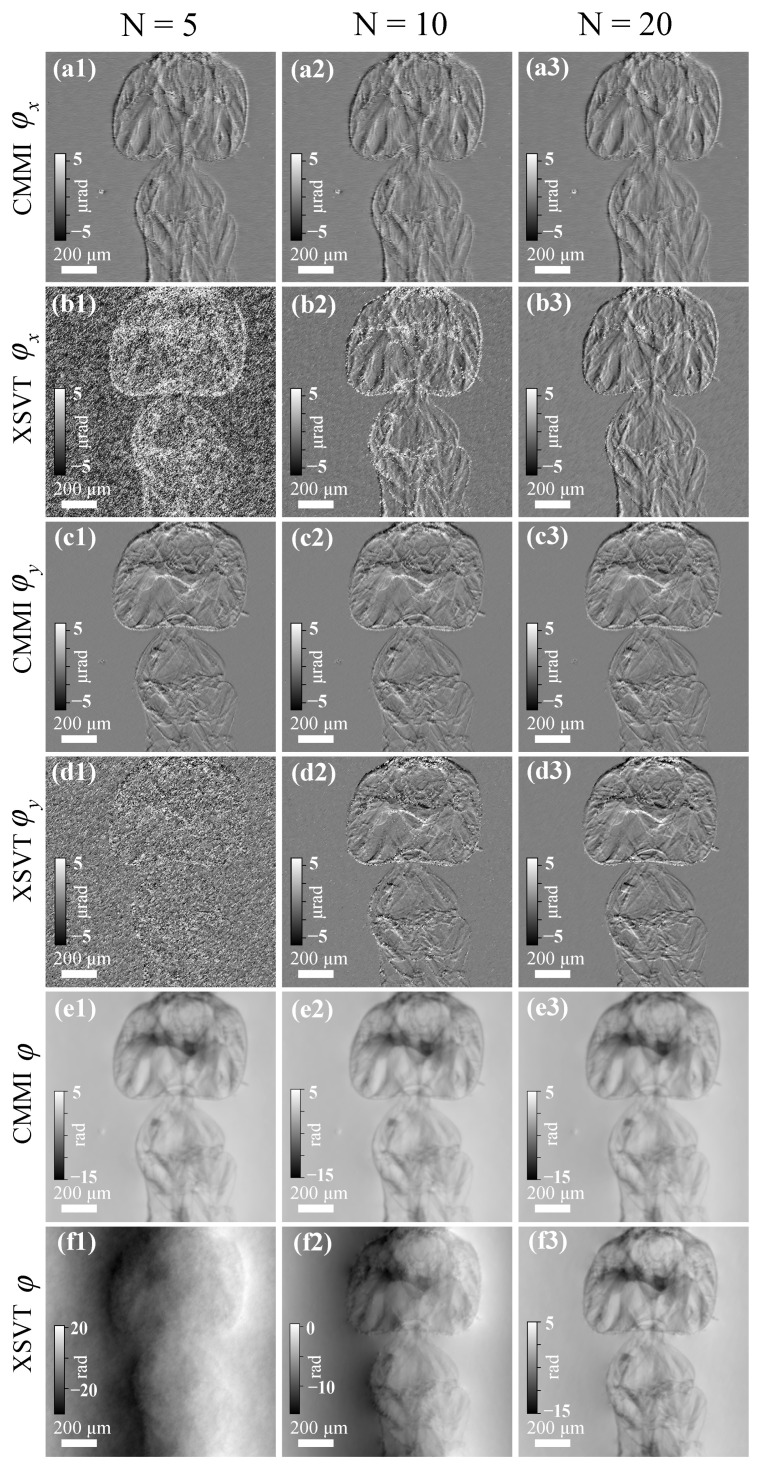
Comparison of scanning CMMI and XSVT results with different numbers of scanning positions (*N* = 5, 10, and 20, for the left, middle, and right columns, respectively). Reconstructed. (**a1**–**a3**) and (**b1**–**b3**) horizontal differential phase, (**c1**–**c3**) and (**d1**–**d3**) vertical differential phase, and (**e1**–**e3**) and (**f1**–**f3**) phase images using scanning CMMI and XSVT, respectively.

Figure 3 shows the reconstructed differential phase and phase images using scanning CMMI and XSVT methods with different *N* values. A constant scanning step size of 3 µm was used for both methods, as shown in Figure 1d. For *N* = 20, both methods can generate high-quality differential phase and phase images, as shown in the right column of Figure 3. The phase image using the XSVT method in Figure 3f3 seems to show a slightly higher contrast than the CMMI phase image in Figure 3e3. However, the reconstructed differential phase images using scanning CMMI in Figure 3(a3,c3) have less noise than the results of XSVT in Figure 3(b3,d3). As *N* decreases to 10, the noise level of XSVT reconstruction increases dramatically, as shown in the middle column of Figure 3. If *N* is further reduced to 5 (left column in Figure 3), the XSVT reconstruction is dominated by noise and provides wrong phase images as expected. In comparison, the scanning CMMI method can provide significantly better reconstruction results even with only five scanning positions. Although the noise of the reconstructed differential phase and phase image by using scanning CMMI does increase for fewer scanning positions, the recovered phase image is still of high quality. The advantage of scanning CMMI in data analysis redundancy indicates that the measurement can be significantly accelerated. This merit can be beneficial for high-resolution tomography, where imaging quality and speed are both important.

### 3.3. High-Resolution Multi-Contrast Tomography

The tomography dataset was collected with 900 projection angles from 0 to 180° with a step size of 0.2°. At each projection angle, scanning CMMI with *N* = 10 was carried out, which has been demonstrated to show accurate 2D reconstruction, as shown in Figure 3. The reference scanning images (without sample) were acquired only once before the sample tomography measurement. The total tomography measurement time was around 4 h with an exposure time of 1 s for each image, which is mainly limited by the X-ray flux and the scanning motor settling time. Two-dimensional transmission, phase, and dark-field images were reconstructed at each projection angle, as described in Section 2.2. The 3D tomography reconstruction was performed using the filtered back-projection (FBP) algorithm with a Ram-Lak filter using the ASTRA toolbox [25,26].

**Figure 4:**
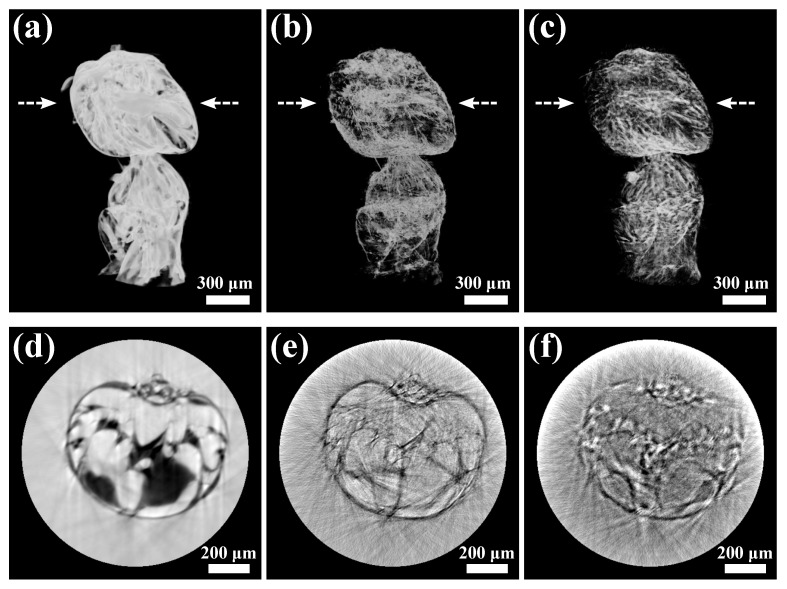
Tomography reconstruction results using scanning CMMI: volume rendering of (**a**) phase, (**b**) dark-field, and (**c**) transmission images of an ant sample. (**d**–**f**) are the reconstruction slices of (**a**–**c**) indicated by the arrow location, respectively.

The reconstructed tomographic results of the ant sample are shown in Figure 4, including the volume rendering of (a) phase, (b) dark-field, and (c) transmission images, and their 2D slices in (d), (e), and (f), respectively. Compared with the transmission 3D volume, the 3D phase volume shows higher contrast thanks to the higher phase sensitivity of X-rays. Again, the 3D dark-field image shows high contrast near the boundaries and edges, which can be helpful complementary information. We should note that there are noticeable artifacts in the tomography reconstruction, indicating a non-optimized tomography setup, such as the rotation motor wobbling and angle errors. Nevertheless, the tomography compatibility of scanning CMMI is successfully demonstrated. By improving the experimental tomography setup, the high quality in 2D image reconstruction shown in Section 3.1 and Section 3.2 could be recovered in the 3D images.

## 4. Conclusions

In summary, scanning CMMI has been introduced for high-resolution and high-sensitivity multi-contrast imaging and tomography. The critical components of scanning CMMI include using a coded phase mask to generate ultra-high-contrast speckle pattern, implementing flexible scanning mode, adapting maximum-likelihood optimization with scanning data, and applying the multi-resolution analysis. Experimental results showed that scanning CMMI could perform simultaneous phase, dark-field, and absorption image reconstruction with higher resolution than single-shot CMMI. Scanning CMMI has superior performance than the correlation-based XSVT in providing similar or higher image contrast with much fewer scanning steps. The choice of the number of steps can adjust the trade-off between resolution and speed, making the technique flexible to different experimental and sample conditions. Finally, multi-contrast tomography was demonstrated using scanning CMMI at each projection angle. Scanning CMMI is a versatile full-field imaging tool that could become the new standard for speckle-based multi-contrast imaging. We anticipate many potential applications, especially in biomedical imaging and tomography.

## Data Availability

Not applicable.
